# 4-(Methyl­amino)benzoic acid

**DOI:** 10.1107/S1600536809038859

**Published:** 2009-09-30

**Authors:** Ísmail Çelik, Mehmet Akkurt, Hacali Necefoğlu, Özgür Aybirdi, Santiago García-Granda

**Affiliations:** a Department of Physics, Faculty of Arts and Sciences, Cumhuriyet University, 58140 Sivas, Turkey; b Department of Physics, Faculty of Arts and Sciences, Erciyes University, 38039 Kayseri, Turkey; c Department of Chemistry, Kafkas University, 63100 Kars, Turkey; d Departamento Química Física y Analítica, Facultad de Química, Universidad Oviedo, C/ Julián Clavería, 8, 33006 Oviedo (Asturias), Spain

## Abstract

The asymmetric unit of the title compound, C_8_H_9_NO_2_, contains three crystallographically independent mol­ecules, which are essentially planar, the carboxyl O atoms deviating by 0.091 (3), 0.101 (2) and 0.164 (3) Å from the mean plane through the non-H atoms. In the crystal, all three mol­ecules form O—H⋯O hydrogen-bonded about inversion centers, forming eight-membered rings with graph-set notation *R*_2_^2^(8). In addition, N—H⋯O hydrogen bonding and C—H⋯π inter­actions reinforce the packing.

## Related literature

For comparison bond-length data in some substituted amino benzoic acid compounds, see: Dzierżawska-Majewska *et al.* (2006 ▶); Smith *et al.* (2007 ▶).
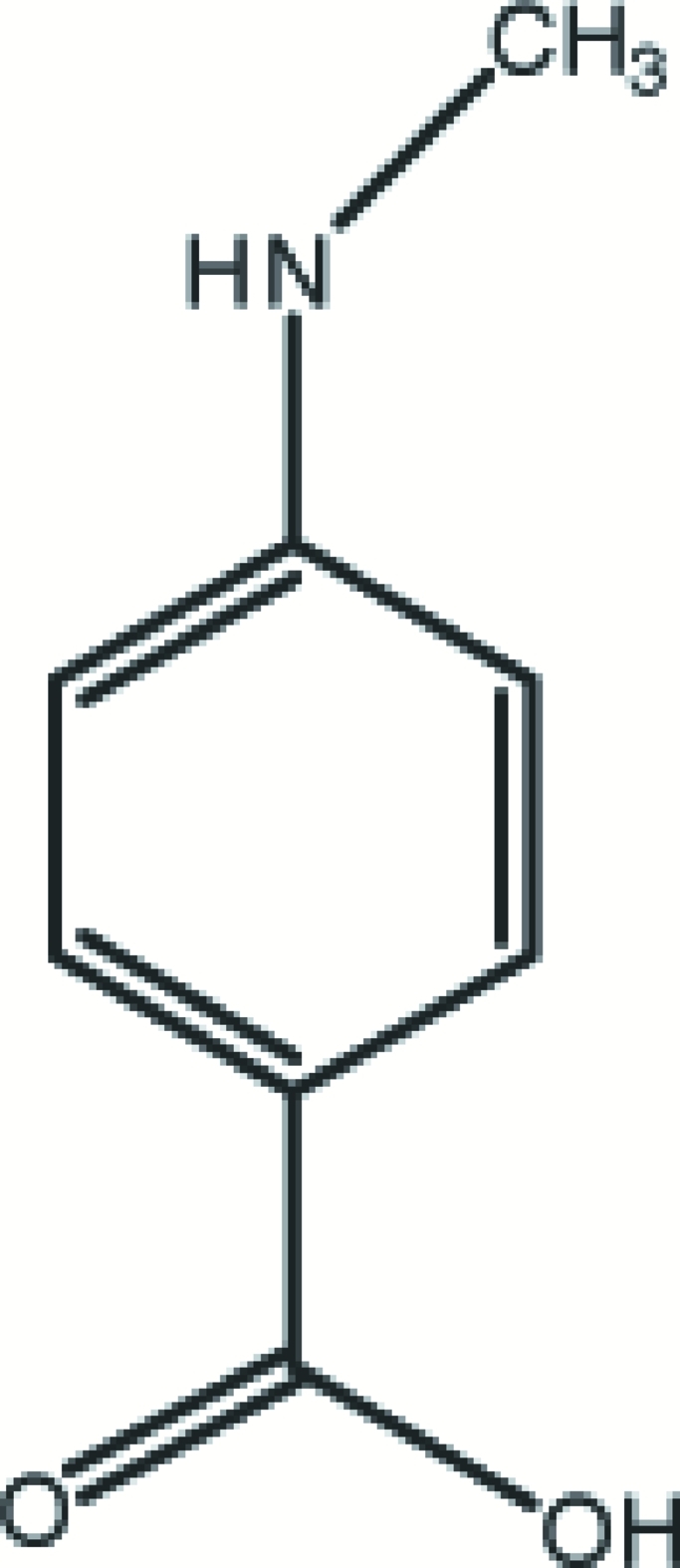


## Experimental

### 

#### Crystal data

C_8_H_9_NO_2_
*M* *_r_* = 151.16
Monoclinic, 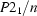

*a* = 5.0456 (3) Å
*b* = 36.339 (2) Å
*c* = 12.6496 (5) Å
β = 96.129 (4)°
*V* = 2306.1 (2) Å^3^
*Z* = 12
Cu *K*α radiation
μ = 0.78 mm^−1^
*T* = 296 K
0.11 × 0.08 × 0.06 mm

#### Data collection

Oxford Diffraction Xcalibur diffractometer with a Ruby Gemini CCD detector
Absorption correction: none
7671 measured reflections
4266 independent reflections
2680 reflections with *I* > 2σ(*I*)
*R*_int_ = 0.043

#### Refinement

*R*[*F* ^2^ > 2σ(*F* ^2^)] = 0.066
*wR*(*F* ^2^) = 0.239
*S* = 1.08
4266 reflections
301 parameters
H-atom parameters constrained
Δρ_max_ = 0.29 e Å^−3^
Δρ_min_ = −0.28 e Å^−3^

### 

Data collection: *CrysAlis Pro* (Oxford Diffraction, 2009 ▶); cell refinement: *CrysAlis Pro*; data reduction: *CrysAlis Pro*; program(s) used to solve structure: *SIR97* (Altomare *et al.*, 1999 ▶); program(s) used to refine structure: *SHELXL97* (Sheldrick, 2008 ▶); molecular graphics: *ORTEP-3 for Windows* (Farrugia, 1999 ▶); software used to prepare material for publication: *WinGX* (Farrugia, 1997 ▶) and *PLATON* (Spek, 2009 ▶).

## Supplementary Material

Crystal structure: contains datablocks global, I. DOI: 10.1107/S1600536809038859/hg2564sup1.cif

Structure factors: contains datablocks I. DOI: 10.1107/S1600536809038859/hg2564Isup2.hkl

Additional supplementary materials:  crystallographic information; 3D view; checkCIF report

## Figures and Tables

**Table 1:** Hydrogen-bond geometry (Å, °)

*D*—H⋯*A*	*D*—H	H⋯*A*	*D*⋯*A*	*D*—H⋯*A*
N1—H1⋯O3	0.86	2.25	3.074 (3)	161
O1—H*O*1⋯O6^i^	0.82	1.86	2.678 (3)	174
N2—H2⋯O6	0.86	2.16	3.003 (3)	168
O4—H*O*4⋯O3^ii^	0.82	1.85	2.661 (3)	171
O5—H*O*5⋯O2^iii^	0.82	1.82	2.627 (4)	170
C24—H24*B*⋯O2^iv^	0.96	2.58	3.305 (4)	132
C8—H8*C*⋯*Cg*1^v^	0.96	2.76	3.564 (4)	142
C16—H16*A*⋯*Cg*2^v^	0.96	2.61	3.482 (4)	151
C24—H24*A*⋯*Cg*3^vi^	0.96	2.70	3.572 (4)	150

Symmetry codes: (i) 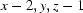; (ii) 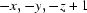; (iii) 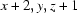; (iv) 
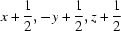
; (v) 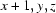; (vi) 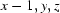. *Cg*1, *Cg*2 and *Cg*3 are the centroids of the C2–C7, C10–C15 and C18–C23 benzene rings, respectively.

**Fig. 1.**
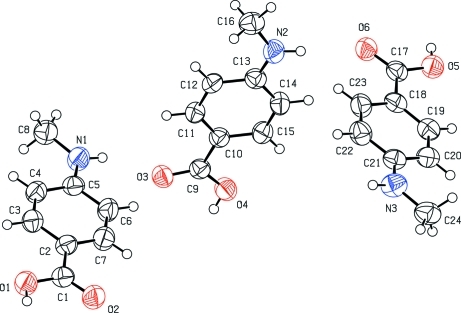
View of the three molecules in the asymmetric unit of the title compound with numbering scheme and thermal ellipsoids drawn at 30% probability.

**Fig. 2.**
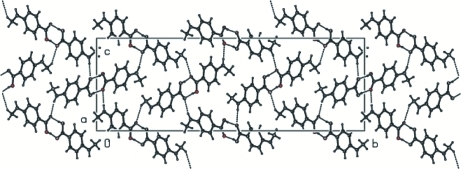
The packing diagram and hydrogen bonding of compound (I) viewed down a axis.

**Fig. 3.**
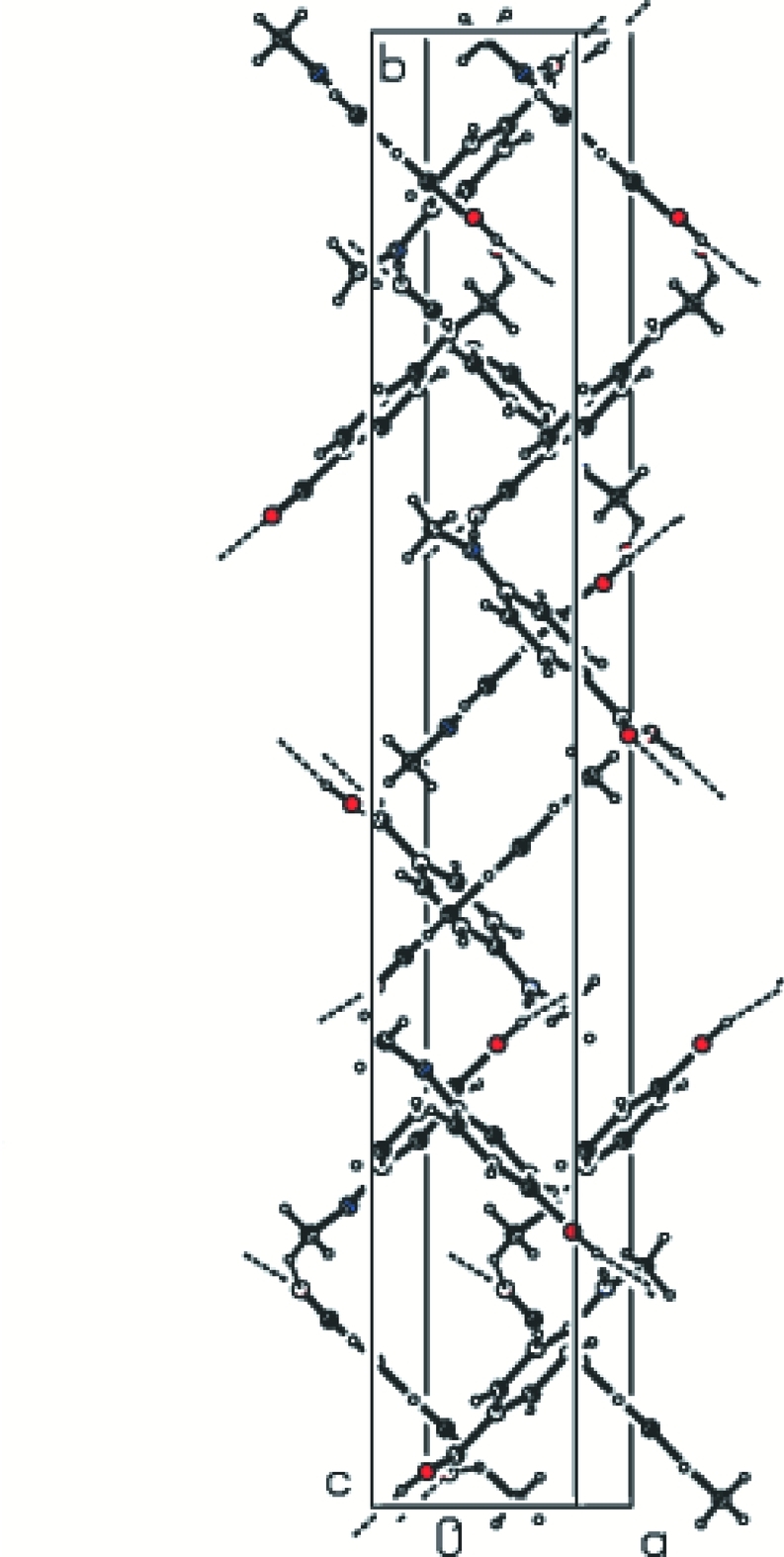
View of the packing of (I) viewed down c axis.

## supplementary crystallographic information

### Comment

Three independent molecules of the title compound (I) in the asymmetric unit are shown in Fig. 1.The bond lengths and angles in the three molecules have normal values. These molecules are not essentially planar (r.m.s. deviations from the mean plane of the non-H atoms are -0.091 (3) Å for atom O1 of the molecule 1, -0.101 (2)Å and 0.164 (3) Å, for atoms O1, O3 and O5 of each molecule, respectively). The dihedral angles between the benzene rings A(C2–C7), B(C10–C17) and C(C18–C23) are A/B = 88.27 (15)°, A/C = 10.63 (16)° and B/C = 89.16 (15)°.

In this structure, the three molecules all form O—H···O hydrogen bonded dimers, between molecules lying about inversion centers, forming eight-membered rings with an *R*_2_^2^(8) motif in graph-set notation (Fig. 2). Further, N—H···O hydrogen bonding and C—H···π interactions reinforces the packing (Table 1, Fig. 3).

### Experimental

4-(Methylamino)benzoic acid (0.60468 g, 0.4 mol) (Aldrich) was added to water of 300 ml. The temperature was kept constant at 343 K and solution stirred in a mixer for 1 h, and then solution was left at room temperature to be crystalline. A day later, colourless crystals formed.

### Refinement

All H atoms were observed in a differences Fourier map. The H atoms in the title compound were placed geometrically [C—H = 0.93 Å for aromatic, C—H = 0.93 Å for methyl, O—H = 0.82 Å for hydroxyl and N—H = 0.86 Å for NH] and refined with *U*_iso_(H) = 1.2 or 1.5*U*_eq_(C, N,*O*), using a riding model. To determine the OH groups correctly, the OH H atoms were geometrically located to both oxygen atoms of the carboxyl group of each molecule, in which their total s.o.f. will be 1. In the final refinement, the values of the s.o.f. 's were determined [0.74 (5) for O1 and 0.26 (5) for O2 in molecule IA, 0.16 (6) for O3 and 0.86 (6) for O4 in molecule IB and 0.75 (5) for O5 and 0.25 (5) for O6 in molecule IC]. Then, *via* the only OH H atoms with the high s.o.f.'s, the refinement process was maintained. Unlike the other two molecules, in the molecule B of the three molecules in the asymmetric unit, the OH H atom is located to the O4(HO4) atom in the opposite direction of the –NHCH_3_ group.

### Crystal data

**Table:**

C_8_H_9_NO_2_	*F*(000) = 960
*M**_r_* = 151.16	*D*_x_ = 1.306 Mg m^−^^3^
Monoclinic, *P*2_1_/*n*	Cu *K*α radiation, λ = 1.54184 Å
Hall symbol: -P 2yn	Cell parameters from 2063 reflections
*a* = 5.0456 (3) Å	θ = 3.5–70.5°
*b* = 36.339 (2) Å	µ = 0.78 mm^−^^1^
*c* = 12.6496 (5) Å	*T* = 296 K
β = 96.129 (4)°	Prism, colourless
*V* = 2306.1 (2) Å^3^	0.11 × 0.08 × 0.06 mm
*Z* = 12	

### Data collection

**Table:**

Oxford Diffraction Xcalibur diffractometer with a Ruby Gemini CCD detector	2680 reflections with *I* > 2σ(*I*)
Radiation source: Enhance (Cu) X-ray Source	*R*_int_ = 0.043
graphite	θ_max_ = 70.9°, θ_min_ = 3.7°
Detector resolution: 10.2673 pixels mm^-1^	*h* = −6→4
ω scans	*k* = −42→43
7671 measured reflections	*l* = −15→14
4266 independent reflections	

### Refinement

**Table:**

Refinement on *F*^2^	Primary atom site location: structure-invariant direct methods
Least-squares matrix: full	Secondary atom site location: difference Fourier map
*R*[*F*^2^ > 2σ(*F*^2^)] = 0.066	Hydrogen site location: inferred from neighbouring sites
*wR*(*F*^2^) = 0.239	H-atom parameters constrained
*S* = 1.08	*w* = 1/[σ^2^(*F*_o_^2^) + (0.1482*P*)^2^] where *P* = (*F*_o_^2^ + 2*F*_c_^2^)/3
4266 reflections	(Δ/σ)_max_ < 0.001
301 parameters	Δρ_max_ = 0.29 e Å^−^^3^
0 restraints	Δρ_min_ = −0.28 e Å^−^^3^

### Special details

**Table:**

Geometry. Bond distances, angles *etc*. have been calculated using the rounded fractional coordinates. All su's are estimated from the variances of the (full) variance-covariance matrix. The cell e.s.d.'s are taken into account in the estimation of distances, angles and torsion angles
Refinement. Refinement on *F*^2^ for ALL reflections except those flagged by the user for potential systematic errors. Weighted *R*-factors *wR* and all goodnesses of fit *S* are based on *F*^2^, conventional *R*-factors *R* are based on *F*, with *F* set to zero for negative *F*^2^. The observed criterion of *F*^2^ > σ(*F*^2^) is used only for calculating -*R*-factor-obs *etc*. and is not relevant to the choice of reflections for refinement. *R*-factors based on *F*^2^ are statistically about twice as large as those based on *F*, and *R*-factors based on ALL data will be even larger.

### Fractional atomic coordinates and isotropic or equivalent isotropic displacement parameters (Å^2^)

**Table:**

	*x*	*y*	*z*	*U*_iso_*/*U*_eq_	
O1	−0.5205 (5)	0.12626 (7)	−0.07946 (17)	0.0620 (8)	
O2	−0.5929 (5)	0.14715 (7)	0.08094 (18)	0.0641 (8)	
N1	0.3153 (6)	0.02834 (8)	0.2055 (2)	0.0596 (10)	
C1	−0.4683 (6)	0.12683 (9)	0.0238 (3)	0.0507 (10)	
C2	−0.2579 (6)	0.10184 (9)	0.0690 (2)	0.0492 (10)	
C3	−0.1092 (7)	0.08070 (9)	0.0043 (3)	0.0539 (10)	
C4	0.0835 (6)	0.05678 (9)	0.0485 (2)	0.0530 (10)	
C5	0.1363 (6)	0.05280 (9)	0.1578 (2)	0.0487 (9)	
C6	−0.0136 (7)	0.07442 (10)	0.2229 (2)	0.0580 (10)	
C7	−0.2032 (7)	0.09828 (10)	0.1783 (3)	0.0598 (11)	
C8	0.4803 (7)	0.00493 (10)	0.1470 (3)	0.0631 (12)	
O3	0.2287 (4)	0.02308 (6)	0.44193 (16)	0.0521 (7)	
O4	0.1669 (5)	0.02371 (7)	0.61435 (17)	0.0598 (8)	
N2	1.0422 (6)	0.14849 (8)	0.6220 (2)	0.0591 (10)	
C9	0.2865 (6)	0.03502 (8)	0.5326 (2)	0.0459 (9)	
C10	0.4885 (6)	0.06362 (8)	0.5575 (2)	0.0444 (9)	
C11	0.6396 (6)	0.07621 (9)	0.4797 (2)	0.0477 (9)	
C12	0.8277 (6)	0.10371 (8)	0.4994 (2)	0.0475 (9)	
C13	0.8671 (6)	0.12006 (8)	0.6004 (2)	0.0470 (9)	
C14	0.7175 (6)	0.10686 (9)	0.6793 (2)	0.0514 (10)	
C15	0.5309 (6)	0.07949 (9)	0.6592 (2)	0.0518 (10)	
C16	1.2134 (7)	0.16264 (10)	0.5479 (3)	0.0610 (11)	
O5	0.9848 (5)	0.18634 (7)	1.01396 (17)	0.0577 (8)	
O6	1.0807 (4)	0.17171 (6)	0.85095 (17)	0.0574 (8)	
N3	0.1885 (6)	0.29613 (8)	0.7547 (2)	0.0628 (10)	
C17	0.9450 (6)	0.18920 (9)	0.9107 (2)	0.0484 (10)	
C18	0.7373 (6)	0.21582 (8)	0.8699 (3)	0.0491 (10)	
C19	0.5713 (6)	0.23242 (9)	0.9371 (3)	0.0516 (10)	
C20	0.3868 (6)	0.25830 (9)	0.8989 (3)	0.0536 (10)	
C21	0.3610 (6)	0.26915 (9)	0.7929 (2)	0.0507 (10)	
C22	0.5231 (7)	0.25154 (9)	0.7247 (3)	0.0585 (11)	
C23	0.7058 (7)	0.22514 (9)	0.7627 (3)	0.0569 (11)	
C24	0.0149 (7)	0.31542 (10)	0.8198 (3)	0.0652 (12)	
H1	0.33120	0.02670	0.27370	0.0720*	
HO1	−0.63770	0.14130	−0.09770	0.0930*	
H3A	−0.14070	0.08280	−0.06930	0.0650*	
H4	0.18040	0.04300	0.00410	0.0630*	
H6	0.01700	0.07240	0.29650	0.0690*	
H7	−0.29810	0.11250	0.22240	0.0720*	
H8A	0.36910	−0.01170	0.10320	0.0950*	
H8B	0.60030	−0.00880	0.19600	0.0950*	
H8C	0.58080	0.01980	0.10290	0.0950*	
H2	1.05010	0.15850	0.68380	0.0710*	
HO4	0.03750	0.01110	0.59310	0.0900*	
H11	0.61370	0.06590	0.41210	0.0570*	
H12	0.92780	0.11140	0.44580	0.0570*	
H14	0.74510	0.11690	0.74720	0.0620*	
H15	0.43270	0.07150	0.71300	0.0620*	
H16A	1.32940	0.14340	0.52840	0.0910*	
H16B	1.31810	0.18250	0.58030	0.0910*	
H16C	1.10710	0.17140	0.48560	0.0910*	
H3	0.18270	0.30200	0.68860	0.0750*	
HO5	1.10800	0.17200	1.03020	0.0870*	
H19	0.58550	0.22590	1.00860	0.0620*	
H20	0.27660	0.26880	0.94510	0.0640*	
H22	0.50720	0.25770	0.65300	0.0700*	
H23	0.80890	0.21350	0.71610	0.0680*	
H24A	−0.09690	0.29800	0.85100	0.0980*	
H24B	−0.09420	0.33250	0.77650	0.0980*	
H24C	0.12050	0.32860	0.87500	0.0980*	

### Atomic displacement parameters (Å^2^)

**Table:**

	*U*^11^	*U*^22^	*U*^33^	*U*^12^	*U*^13^	*U*^23^
O1	0.0639 (15)	0.0705 (16)	0.0507 (12)	0.0160 (12)	0.0017 (11)	0.0032 (11)
O2	0.0620 (14)	0.0708 (16)	0.0587 (13)	0.0156 (12)	0.0035 (11)	−0.0050 (12)
N1	0.0614 (16)	0.0710 (19)	0.0462 (14)	0.0174 (14)	0.0046 (12)	0.0048 (13)
C1	0.0458 (16)	0.0541 (18)	0.0525 (17)	0.0004 (14)	0.0061 (14)	0.0050 (15)
C2	0.0448 (16)	0.0522 (18)	0.0513 (16)	0.0043 (13)	0.0080 (13)	0.0014 (14)
C3	0.0555 (18)	0.061 (2)	0.0451 (15)	0.0011 (15)	0.0054 (14)	0.0020 (15)
C4	0.0520 (17)	0.058 (2)	0.0495 (17)	0.0081 (15)	0.0079 (14)	−0.0025 (15)
C5	0.0414 (15)	0.0537 (18)	0.0513 (16)	−0.0015 (13)	0.0062 (13)	0.0029 (14)
C6	0.0600 (19)	0.071 (2)	0.0428 (15)	0.0112 (17)	0.0052 (14)	0.0001 (15)
C7	0.062 (2)	0.066 (2)	0.0512 (18)	0.0112 (17)	0.0053 (15)	−0.0032 (16)
C8	0.064 (2)	0.065 (2)	0.060 (2)	0.0114 (17)	0.0052 (16)	0.0013 (17)
O3	0.0541 (12)	0.0559 (13)	0.0456 (11)	−0.0049 (10)	0.0020 (9)	0.0007 (10)
O4	0.0584 (14)	0.0741 (17)	0.0470 (12)	−0.0168 (12)	0.0063 (10)	0.0013 (11)
N2	0.0625 (17)	0.0565 (17)	0.0594 (16)	−0.0091 (13)	0.0113 (13)	−0.0141 (13)
C9	0.0434 (15)	0.0498 (17)	0.0443 (15)	0.0059 (13)	0.0034 (13)	0.0060 (13)
C10	0.0426 (15)	0.0470 (16)	0.0434 (15)	0.0029 (12)	0.0041 (12)	0.0026 (13)
C11	0.0485 (16)	0.0497 (17)	0.0451 (15)	0.0030 (13)	0.0061 (13)	−0.0054 (13)
C12	0.0429 (15)	0.0536 (18)	0.0477 (16)	−0.0006 (13)	0.0127 (13)	−0.0019 (14)
C13	0.0446 (15)	0.0448 (16)	0.0509 (16)	0.0028 (12)	0.0024 (13)	0.0003 (13)
C14	0.0584 (18)	0.0542 (19)	0.0417 (15)	−0.0022 (15)	0.0057 (14)	−0.0034 (14)
C15	0.0549 (18)	0.0575 (19)	0.0438 (15)	−0.0017 (15)	0.0092 (13)	0.0025 (14)
C16	0.0547 (19)	0.056 (2)	0.073 (2)	−0.0041 (16)	0.0097 (17)	0.0014 (17)
O5	0.0566 (14)	0.0633 (15)	0.0522 (12)	0.0102 (11)	0.0010 (10)	0.0001 (11)
O6	0.0572 (13)	0.0593 (14)	0.0545 (12)	0.0078 (11)	0.0009 (10)	−0.0070 (11)
N3	0.0596 (17)	0.0620 (18)	0.0664 (17)	0.0113 (14)	0.0054 (14)	0.0139 (15)
C17	0.0451 (16)	0.0451 (17)	0.0535 (17)	−0.0046 (13)	−0.0010 (14)	−0.0052 (14)
C18	0.0428 (16)	0.0458 (17)	0.0568 (17)	−0.0044 (13)	−0.0030 (14)	−0.0016 (14)
C19	0.0511 (17)	0.0518 (18)	0.0510 (17)	−0.0050 (14)	0.0017 (14)	0.0050 (14)
C20	0.0451 (16)	0.0545 (19)	0.0606 (18)	−0.0004 (14)	0.0030 (14)	−0.0015 (15)
C21	0.0442 (16)	0.0481 (17)	0.0591 (18)	−0.0035 (13)	0.0022 (14)	0.0003 (14)
C22	0.064 (2)	0.057 (2)	0.0528 (18)	0.0046 (16)	−0.0019 (16)	0.0018 (15)
C23	0.0602 (19)	0.056 (2)	0.0529 (18)	0.0052 (15)	−0.0007 (15)	−0.0076 (15)
C24	0.055 (2)	0.059 (2)	0.080 (2)	0.0056 (16)	−0.0002 (18)	0.0012 (18)

### Geometric parameters (Å, °)

**Table:**

O1—C1	1.305 (4)	C8—H8C	0.9600
O2—C1	1.249 (4)	C8—H8A	0.9600
O1—HO1	0.8200	C9—C10	1.466 (4)
O3—C9	1.232 (3)	C10—C11	1.385 (4)
O4—C9	1.318 (4)	C10—C15	1.405 (4)
O4—HO4	0.8200	C11—C12	1.382 (4)
O5—C17	1.304 (3)	C12—C13	1.404 (4)
O6—C17	1.247 (4)	C13—C14	1.399 (4)
O5—HO5	0.8200	C14—C15	1.375 (4)
N1—C8	1.448 (5)	C11—H11	0.9300
N1—C5	1.361 (4)	C12—H12	0.9300
N1—H1	0.8600	C14—H14	0.9300
N2—C16	1.436 (5)	C15—H15	0.9300
N2—C13	1.368 (4)	C16—H16C	0.9600
N2—H2	0.8600	C16—H16B	0.9600
N3—C24	1.446 (5)	C16—H16A	0.9600
N3—C21	1.365 (4)	C17—C18	1.478 (4)
N3—H3	0.8600	C18—C23	1.390 (5)
C1—C2	1.466 (4)	C18—C19	1.393 (5)
C2—C3	1.398 (5)	C19—C20	1.374 (5)
C2—C7	1.387 (5)	C20—C21	1.390 (5)
C3—C4	1.378 (5)	C21—C22	1.405 (5)
C4—C5	1.387 (4)	C22—C23	1.381 (5)
C5—C6	1.414 (4)	C19—H19	0.9300
C6—C7	1.368 (5)	C20—H20	0.9300
C3—H3A	0.9300	C22—H22	0.9300
C4—H4	0.9300	C23—H23	0.9300
C6—H6	0.9300	C24—H24A	0.9600
C7—H7	0.9300	C24—H24B	0.9600
C8—H8B	0.9600	C24—H24C	0.9600
			
O1···O6^i^	2.678 (3)	C20···H24A^viii^	3.0900
O1···C17^ii^	3.290 (4)	C20···H24A	2.8400
O2···C24^iii^	3.305 (4)	C20···H16C^xv^	2.9500
O2···O5^i^	2.627 (4)	C20···H24C	2.8900
O2···C17^i^	3.367 (4)	C21···H24A^viii^	2.9500
O2···N3^iii^	3.229 (4)	C22···H24A^viii^	2.9000
O3···C9^iv^	3.221 (4)	C23···H24A^viii^	3.0000
O3···C9^v^	3.391 (4)	C24···H7^ix^	3.0900
O3···N1	3.074 (3)	C24···H20	2.5800
O3···O4^v^	2.661 (3)	H1···O3	2.2500
O4···O3^v^	2.661 (3)	H1···H6	2.3300
O5···C1^vi^	3.159 (4)	H1···H11	2.5700
O5···C2^vi^	3.405 (4)	HO1···O6^i^	1.8600
O5···O2^vii^	2.627 (4)	HO1···C17^i^	2.7400
O6···N2	3.003 (3)	HO1···HO5^i^	2.4400
O6···C19^viii^	3.407 (4)	H2···O6	2.1600
O6···C1^vii^	3.400 (4)	H2···H23	2.4000
O6···O1^vii^	2.678 (3)	H2···H14	2.3600
O1···H14^ii^	2.7100	HO4···HO4^v^	2.4800
O1···H3A	2.4800	HO4···O3^v^	1.8500
O2···HO5^i^	1.8200	HO4···C9^v^	2.7300
O2···H24B^iii^	2.5800	H3···O5^iii^	2.8500
O2···H7	2.5400	H3···H19^iii^	2.4900
O2···H3^iii^	2.6100	H3···H22	2.3700
O3···H6	2.7100	H3···O2^ix^	2.6100
O3···H11	2.5500	HO5···HO1^vii^	2.4400
O3···H1	2.2500	HO5···O2^vii^	1.8200
O3···HO4^v^	1.8500	HO5···C1^vii^	2.7000
O4···H15	2.4500	H3A···O1	2.4800
O4···H8B^iv^	2.6100	H4···H8C	2.4100
O5···H3^ix^	2.8500	H4···H8A	2.4800
O5···H22^ix^	2.6800	H4···C8	2.6200
O5···H19	2.4700	H6···O3	2.7100
O6···H23	2.5700	H6···H1	2.3300
O6···H2	2.1600	H6···C12^x^	3.0500
O6···HO1^vii^	1.8600	H6···H12^x^	2.4400
O6···H14	2.8400	H7···O2	2.5400
N1···O3	3.074 (3)	H7···C24^iii^	3.0900
N2···O6	3.003 (3)	H8A···C4	2.9200
N3···O2^ix^	3.229 (4)	H8A···C3^xiii^	3.0800
N2···H23	2.9500	H8A···H4	2.4800
C1···O6^i^	3.400 (4)	H8B···O4^iv^	2.6100
C1···C4^x^	3.441 (4)	H8B···H15^iv^	2.5700
C1···O5^ii^	3.159 (4)	H8C···H4	2.4100
C1···C17^ii^	3.489 (4)	H8C···C4^viii^	3.0100
C2···O5^ii^	3.405 (4)	H8C···C4	2.8600
C4···C1^viii^	3.441 (4)	H8C···C5^viii^	3.0600
C9···C9^iv^	3.490 (4)	H8C···C3^viii^	3.0500
C9···O3^iv^	3.221 (4)	H11···H1	2.5700
C9···C11^x^	3.588 (4)	H11···O3	2.5500
C9···C12^x^	3.400 (4)	H12···H16C	2.3900
C9···O3^v^	3.391 (4)	H12···C16	2.6100
C11···C9^viii^	3.588 (4)	H12···H16A	2.4700
C12···C9^viii^	3.400 (4)	H12···H6^viii^	2.4400
C14···C16^x^	3.527 (5)	H14···O1^vi^	2.7100
C16···C14^viii^	3.527 (5)	H14···H2	2.3600
C16···C24^xi^	3.495 (5)	H14···O6	2.8400
C17···C19^viii^	3.513 (4)	H15···O4	2.4500
C17···C20^viii^	3.372 (4)	H15···H8B^iv^	2.5700
C17···C1^vi^	3.489 (4)	H16A···C11^viii^	3.0000
C17···O2^vii^	3.367 (4)	H16A···C12	2.9000
C17···O1^vi^	3.290 (4)	H16A···C10^viii^	3.0200
C19···C17^x^	3.513 (4)	H16A···C13^viii^	2.9000
C19···O6^x^	3.407 (4)	H16A···C14^viii^	2.9100
C20···C17^x^	3.372 (4)	H16A···C12^viii^	2.9600
C22···C24^viii^	3.515 (5)	H16A···H12	2.4700
C24···C22^x^	3.515 (5)	H16A···C15^viii^	2.9700
C24···O2^ix^	3.305 (4)	H16C···C20^xiv^	2.9500
C24···C16^xii^	3.495 (5)	H16C···H12	2.3900
C1···HO5^i^	2.7000	H16C···C12	2.8500
C3···H8C^x^	3.0500	H19···O5	2.4700
C3···H8A^xiii^	3.0800	H19···H3^ix^	2.4900
C4···H8A	2.9200	H20···C24	2.5800
C4···H8C	2.8600	H20···H24A	2.3700
C4···H8C^x^	3.0100	H20···H24C	2.4500
C5···H8C^x^	3.0600	H22···H3	2.3700
C8···H4	2.6200	H22···O5^iii^	2.6800
C9···HO4^v^	2.7300	H23···O6	2.5700
C10···H16A^x^	3.0200	H23···N2	2.9500
C11···H16A^x^	3.0000	H23···H2	2.4000
C12···H16C	2.8500	H24A···C20^x^	3.0900
C12···H6^viii^	3.0500	H24A···C20	2.8400
C12···H16A	2.9000	H24A···C21^x^	2.9500
C12···H16A^x^	2.9600	H24A···C22^x^	2.9000
C12···H24C^xiv^	3.0400	H24A···C23^x^	3.0000
C13···H16A^x^	2.9000	H24A···H20	2.3700
C14···H16A^x^	2.9100	H24B···O2^ix^	2.5800
C15···H16A^x^	2.9700	H24C···C20	2.8900
C16···H12	2.6100	H24C···H20	2.4500
C17···HO1^vii^	2.7400	H24C···C12^xv^	3.0400
			
C1—O1—HO1	109.00	C12—C13—C14	118.0 (3)
C9—O4—HO4	109.00	N2—C13—C14	120.2 (2)
C17—O5—HO5	109.00	N2—C13—C12	121.8 (3)
C5—N1—C8	123.2 (3)	C13—C14—C15	121.8 (2)
C5—N1—H1	118.00	C10—C15—C14	120.0 (3)
C8—N1—H1	118.00	C12—C11—H11	119.00
C13—N2—C16	124.1 (3)	C10—C11—H11	119.00
C16—N2—H2	118.00	C11—C12—H12	120.00
C13—N2—H2	118.00	C13—C12—H12	120.00
C21—N3—C24	123.3 (3)	C13—C14—H14	119.00
C24—N3—H3	118.00	C15—C14—H14	119.00
C21—N3—H3	118.00	C10—C15—H15	120.00
O1—C1—C2	116.2 (3)	C14—C15—H15	120.00
O1—C1—O2	121.9 (3)	N2—C16—H16B	109.00
O2—C1—C2	121.9 (3)	N2—C16—H16C	109.00
C1—C2—C7	120.4 (3)	H16A—C16—H16B	110.00
C3—C2—C7	118.1 (3)	H16A—C16—H16C	109.00
C1—C2—C3	121.6 (3)	N2—C16—H16A	109.00
C2—C3—C4	120.6 (3)	H16B—C16—H16C	110.00
C3—C4—C5	121.5 (3)	O6—C17—C18	122.5 (3)
N1—C5—C4	123.9 (3)	O5—C17—C18	115.4 (3)
N1—C5—C6	118.4 (2)	O5—C17—O6	122.1 (3)
C4—C5—C6	117.7 (3)	C17—C18—C19	121.4 (3)
C5—C6—C7	120.4 (3)	C17—C18—C23	120.2 (3)
C2—C7—C6	121.8 (3)	C19—C18—C23	118.4 (3)
C4—C3—H3A	120.00	C18—C19—C20	120.7 (3)
C2—C3—H3A	120.00	C19—C20—C21	121.6 (3)
C3—C4—H4	119.00	N3—C21—C22	120.1 (3)
C5—C4—H4	119.00	N3—C21—C20	122.4 (3)
C7—C6—H6	120.00	C20—C21—C22	117.5 (3)
C5—C6—H6	120.00	C21—C22—C23	120.9 (3)
C6—C7—H7	119.00	C18—C23—C22	120.8 (3)
C2—C7—H7	119.00	C18—C19—H19	120.00
H8A—C8—H8C	109.00	C20—C19—H19	120.00
H8A—C8—H8B	110.00	C19—C20—H20	119.00
H8B—C8—H8C	109.00	C21—C20—H20	119.00
N1—C8—H8A	109.00	C21—C22—H22	120.00
N1—C8—H8C	110.00	C23—C22—H22	119.00
N1—C8—H8B	109.00	C18—C23—H23	120.00
O3—C9—C10	122.7 (2)	C22—C23—H23	120.00
O3—C9—O4	122.6 (3)	N3—C24—H24A	109.00
O4—C9—C10	114.8 (2)	N3—C24—H24B	109.00
C11—C10—C15	118.3 (3)	N3—C24—H24C	109.00
C9—C10—C15	121.5 (3)	H24A—C24—H24B	110.00
C9—C10—C11	120.3 (2)	H24A—C24—H24C	110.00
C10—C11—C12	122.0 (2)	H24B—C24—H24C	109.00
C11—C12—C13	119.9 (3)		
			
C8—N1—C5—C4	3.1 (5)	C9—C10—C15—C14	−178.5 (3)
C8—N1—C5—C6	−179.7 (3)	C9—C10—C11—C12	178.5 (3)
C16—N2—C13—C14	176.8 (3)	C15—C10—C11—C12	−0.2 (5)
C16—N2—C13—C12	−4.4 (5)	C11—C10—C15—C14	0.3 (5)
C24—N3—C21—C20	−1.0 (5)	C10—C11—C12—C13	−0.9 (5)
C24—N3—C21—C22	179.8 (3)	C11—C12—C13—C14	1.9 (4)
O2—C1—C2—C7	4.4 (5)	C11—C12—C13—N2	−177.0 (3)
O1—C1—C2—C7	−174.7 (3)	N2—C13—C14—C15	177.0 (3)
O2—C1—C2—C3	−176.4 (3)	C12—C13—C14—C15	−1.9 (5)
O1—C1—C2—C3	4.6 (5)	C13—C14—C15—C10	0.8 (5)
C7—C2—C3—C4	0.8 (5)	O5—C17—C18—C19	−8.9 (4)
C1—C2—C7—C6	178.1 (3)	O5—C17—C18—C23	170.4 (3)
C1—C2—C3—C4	−178.5 (3)	O6—C17—C18—C19	173.9 (3)
C3—C2—C7—C6	−1.2 (5)	O6—C17—C18—C23	−6.9 (5)
C2—C3—C4—C5	0.1 (5)	C17—C18—C19—C20	177.2 (3)
C3—C4—C5—C6	−0.5 (5)	C23—C18—C19—C20	−2.1 (5)
C3—C4—C5—N1	176.8 (3)	C17—C18—C23—C22	−176.2 (3)
C4—C5—C6—C7	0.0 (5)	C19—C18—C23—C22	3.1 (5)
N1—C5—C6—C7	−177.4 (3)	C18—C19—C20—C21	−0.8 (5)
C5—C6—C7—C2	0.9 (5)	C19—C20—C21—N3	−176.6 (3)
O4—C9—C10—C11	176.1 (3)	C19—C20—C21—C22	2.6 (5)
O4—C9—C10—C15	−5.2 (4)	N3—C21—C22—C23	177.6 (3)
O3—C9—C10—C15	173.2 (3)	C20—C21—C22—C23	−1.7 (5)
O3—C9—C10—C11	−5.5 (5)	C21—C22—C23—C18	−1.2 (5)

Symmetry codes: (i) *x*−2, *y*, *z*−1; (ii) *x*−1, *y*, *z*−1; (iii) *x*−1/2, −*y*+1/2, *z*−1/2; (iv) −*x*+1, −*y*, −*z*+1; (v) −*x*, −*y*, −*z*+1; (vi) *x*+1, *y*, *z*+1; (vii) *x*+2, *y*, *z*+1; (viii) *x*+1, *y*, *z*; (ix) *x*+1/2, −*y*+1/2, *z*+1/2; (x) *x*−1, *y*, *z*; (xi) *x*+3/2, −*y*+1/2, *z*−1/2; (xii) *x*−3/2, −*y*+1/2, *z*+1/2; (xiii) −*x*, −*y*, −*z*; (xiv) *x*+1/2, −*y*+1/2, *z*−1/2; (xv) *x*−1/2, −*y*+1/2, *z*+1/2.

### Hydrogen-bond geometry (Å, °)

**Table:**

*D*—H···*A*	*D*—H	H···*A*	*D*···*A*	*D*—H···*A*
N1—H1···O3	0.86	2.25	3.074 (3)	161
O1—HO1···O6^i^	0.82	1.86	2.678 (3)	174
N2—H2···O6	0.86	2.16	3.003 (3)	168
O4—HO4···O3^v^	0.82	1.85	2.661 (3)	171
O5—HO5···O2^vii^	0.82	1.82	2.627 (4)	170
C24—H24B···O2^ix^	0.96	2.58	3.305 (4)	132
C8—H8C···Cg1^viii^	0.96	2.76	3.564 (4)	142
C16—H16A···Cg2^viii^	0.96	2.61	3.482 (4)	151
C24—H24A···Cg3^x^	0.96	2.70	3.572 (4)	150

Symmetry codes: (i) *x*−2, *y*, *z*−1; (v) −*x*, −*y*, −*z*+1; (vii) *x*+2, *y*, *z*+1; (ix) *x*+1/2, −*y*+1/2, *z*+1/2; (viii) *x*+1, *y*, *z*; (x) *x*−1, *y*, *z*.
